# Factors influencing the efficacy of invisalign in molar distalization and tooth movement

**DOI:** 10.3389/fbioe.2023.1215169

**Published:** 2023-10-25

**Authors:** Xiaowen Chen, Ying Shi, Jieying Yuan, Ye Li, Weican Chen

**Affiliations:** Department of Stomatology, Baoding First Central Hospital, Baoding, China

**Keywords:** invisible orthodontic appliance, molar distalization, clinical efficacy, invisalign, tooth movement

## Abstract

**Introduction:** The work aims to establish and analyze the factors influencing the efficacy of Invisalign in molar distalization and tooth movement. Objectives of the study: 1) identify factors contributing to molar distalization and tooth movement; 2) analyze the effectiveness of Invisalign technology in molar distalization and tooth movement.

**Methods:** The study was conducted in 2020–2022 in Baoding (PRC) based on Baoding’s first central hospital. Forty patients (mean age 28.5 ± 1.5 years, 18–35 years; 20 women and 20 men) participated in the study.

**Results:** All patients had mild to moderate degrees of tooth crowding, with an angle class II malocclusion, as well as maxillary third molars. Before and after the therapy, the condition of the alveolar bone, soft tissues, and facial height were measured. Fixation of the anterior teeth was performed. Calculations of the distal molar movement were performed. For orthodontic procedures, the second M of the upper jaw were moved, and then the first molars were moved.

**Discussion:** Distalization of the upper molars was found to be an effective movement, with an efficiency of about 83% when vertical rectangular attachments were used. The first molar was moved distally by 2.85 mm without significant tilt or movement in the vertical plane.

## 1 Introduction

An ever-increasing trend in today’s reality is for patients to visit a dentist; this is due to the aesthetics of the result and the fact that such visits become more and more comfortable. Particularly, patients with malocclusion expect to get a beautiful smile for themselves from an orthodontist, but without significant changes in their lifestyle. As a result of these demands and expectations within the field of medicine, there is an ongoing pursuit of novel approaches to address various dental and jaw anomalies. These endeavours encompass diverse aspects, such as aesthetics, practicality, and precise outcomes (Nanda, 2005). While braces used to be the most common, aligners are now growing in popularity. Aligners are called transparent mouthguards that are not visible to other people. Aligners are manufactured based on 3D technology. In addition, the technique of positioning vestibular braces using 3D images is quite common (Phulari, 2013). This technique reduces the percentage of errors as well as possible complications. Thus, 3D technology is quite widely used in modern dentistry and orthodontics.

The prevalence of braces due to their unaesthetic appearance has been the reason for the frequent rejection of dental services. In addition, if the appliance is fixed, a patient needs to be very careful with their hygiene and even use additional means to prevent the development of cavities and other dental health problems. Since most often the recommendations of a dentist are not fully followed by patients, it is necessary to choose such a type of orthodontic appliance so that it does not interfere with the observance of basic hygiene procedures related to the oral cavity (Macrì et al., 2022). In this regard, the development of new technologies, which began in the late 20th century, took another 20 years, and eventually came in the form of aligners, by the name of the company Align Technology, Inc. (Kuo and Miller, 2003). The essence of the invention was to exert pressure on the tooth surface, resulting in its movement to the correct position. Aligners are removable and consist of a translucent biopolymer. A patient is required to replace one removable aligner every 14 days with another that is slightly different in shape. Each of the removable aligners is characterized by a different movement, which in total leads to the desired result. It should be noted that aligners are only used for adults (Al-Nadawi et al., 2021). This is because it is impossible to predict the exact course of therapy and the use of a set of aligners for children whose jaws are growing. Aligners are employed in cases of orthodontic relapse after treatment and are also utilized for addressing various dental issues such as crowded teeth, deep malocclusion, crossbite, and open bites. According to some reports, aligners cannot be used for the treatment of high levels of crowding, or complex orthodontic surgery (Phulari, 2013). In 2010, aligners were improved, making it possible to expand the treatment range. Aligners have fasteners that make it possible to move teeth according to a predetermined therapy plan (Sachdev et al., 2021).

### 1.1 Literature review

Presently, aligners provide the ability to treat both crowding and the rotation of molars, premolars, and incisors. In addition, intrusion and distalization of teeth on both jaws can be performed with aligners (Macrì et al., 2022). Aligners therapy can be divided into several stages. These include methods that allow for an effective diagnosis of the condition of the teeth and jaws in the initial stage, based on which a treatment plan is drawn up. In the second stage, impressions are taken, and plaster or virtual models are used. The models are printed using a 3D printer. In the third stage, a setup model is made on a personal computer using specialized software. The treatment plan is linked to a video simulating the therapy. Then, in the fourth step, aligners are produced in two ways, either on a 3D printer or with the Dynamic Physical Model. In the last, fifth step, the clinical management of a patient is performed. The creation of the digital model is associated with silicone impressions and recorders that establish the occlusion parameters. The model in virtual space can be rotated and viewed in different sections (Jindal et al., 2020; Lümkemann et al., 2023). In some cases, an additional option is offered based on the results of a computed tomography scan, which can be used to see how the roots of the teeth move (StarSmile). This makes it possible to predict the distribution of bone tissue during therapy. The diagnosis makes it possible to identify the relationship between the parameters of occlusion of the jaw arches in the case of pathology, and it also becomes possible to measure the value of the sagittal curve (Henrikson et al., 2001; Lepidi et al., 2023).

Based on the above, the advantages of using aligners are a doctor’s ability to show a patient all the stages of treatment on specific 3D models; transparency and aesthetics of the aligners; preservation of quality of life without complications at work or home due to the appearance compared to the braces (Sauer et al., 2023). In addition, since the aligners are removable, they do not interfere with basic oral hygiene. Aligners can be cleaned with toothpaste and a standard toothbrush (Levrini et al., 2015). They do not affect eating habits, do not interfere with swallowing and chewing; they also do not cause oral trauma (Chen et al., 2022). Standard orthodontic appliances have been reported to result in a high incidence of oral mucosal ulcers due to the presence of metal elements (Rahilly and Price, 2003). For patients with aligners, the increase in pain within a week of the start of therapy is significantly lower than for patients with braces. Patients with aligners are less likely to use medications such as analgesics (Macrì et al., 2022). The nature of the pain differs as well: it is more tolerable in patients with aligners (they might feel some pressure), while in patients with braces, the pain is throbbing. The plastic used in the aligners is hypoallergenic, allowing patients with metal allergies to be treated as well. Patients with aligners visit the orthodontist less frequently (Jindal et al., 2020). Aligners are individually and precisely made for each patient using the Dynamic Physical Model. A study comparing the performance of the two technologies showed that patients with 3D-printed aligners lagged behind those with Dynamic Physical Model aligners. In some cases, additional teeth impressions had to be made because 3D printing did not always fit the aligner precisely to the teeth (Lümkemann et al., 2023).

However, aligners also have their disadvantages. They include a high price, a smaller list of indications for use compared with braces, and possible complications from improper processing of scanning results in a specialized computer program. Therefore, pre-treatment is significantly important for treatment with aligners. In the case of a strong tooth deviation, complications are possible because the boundaries of the alveolar processes are displaced, and the muscle tone changes (Lou et al., 2021). A dentist has less control over teeth movement during treatment with aligners; it is more up to the patient. In particular, the wearing of aligners should be no less than 22 h out of 24, their removal is allowed only during meals and dental hygiene procedures (Sauer et al., 2023). The break time should not exceed 20 min. For some diagnoses, the results of aligners are slower than the braces. Initially, it is rather difficult to speak intelligibly with aligners. If an aligner is not put on correctly, it may crack, and gum trauma may occur. There are two solutions to a ruptured aligner: make a new one or put the next one in line. Treatment with an aligner involves no signs of tooth decay and a completely sanitized mouth. In the case of an exacerbation of caries, the aligner may not fit over a restored or filled tooth (Sauer et al., 2023).

### 1.2 Problem statement

Contemporary technologies, such as aligners, have become widely prevalent in orthodontic practice; however, their application remains contentious in certain cases. The following points are among the aspects of the debate:

Challenging or atypical cases: Aligners are typically effective in treating mild to moderate cases of occlusal anomalies. However, in complex or non-standard cases, such as severe dental or skeletal deformities, the effectiveness of aligners may be limited. In such situations, orthodontists may prefer traditional braces, which provide more precise control over tooth movement.

Patient cooperation: For successful treatment with aligners, active patient cooperation with the healthcare provider and adherence to all recommendations regarding device usage, wear schedule, and regular visits to the orthodontist are essential. Failure to comply with the prescribed wear regimen or lack of patient cooperation may lead to less predictable treatment outcomes, potentially causing disputes between the healthcare provider and the patient.

Comparison with traditional braces: Some orthodontists and patients may debate over the preferable treatment method: aligners or traditional braces. Each approach possesses its advantages and drawbacks, and the selection depends on the specific situation and patient preferences.

Treatment efficacy: Despite numerous studies and evidence supporting the effectiveness of aligners in treating specific cases, some specialists may cast doubt upon their outcomes, particularly when comparing them to the results achieved through traditional braces.

Cost: Treatment with aligners may incur higher expenses compared to traditional braces. Issues about the pricing and accessibility of such treatment may also be a source of contention.

Furthermore, the investigation of factors determining the feasibility of utilizing the Invisalign system for molar distalization remains understudied. Hence, the relevance of conducting the present study is beyond doubt. The work aims to establish and analyze the factors influencing the efficacy of Invisalign in molar distalization and tooth movement. Study objectives.

a) compare the rates of volume, speed, and time taken for molar distalization during treatment with braces and aligners (both with microimplants);
b) establish the stages of biomechanical changes occurring during molar distalization during the use of braces and Invisalign aligners;
c) analyze the state of the oral cavity in patients with braces and with aligners;
d) establish the optimal algorithm during treatment with Invisalign aligners with microimplants.

## 2 Materials and methods

### 2.1 Sample

The study was conducted in 2020–2022 at the First Central Hospital in Baoding, China. This study belongs to the category of observational (non-controlled) research. Forty patients participated in the study, of whom 20 were men. The mean age of the patients was 28.5 ± 1.5 years, ranging from 18 to 35 years. The indications of the patients for the use of aligners were as follows: the presence of mild to moderate crowding of teeth and third molars in the upper jaw. Before treatment, all patients underwent molar extraction (maxillary third molars, left and right, localization–upper jaw). Invisalign aligners were used for treatment. The statistical power of the test was approximately 85% at a significance level of α = 0.05.

### 2.2 Inclusion and non-inclusion criteria for the study

The criteria for inclusion of a patient in the study were.

a) Appropriateness of age;
b) Availability of an appropriate diagnosis;
c) A patient had not had any orthodontic treatment before therapy;
d) The jaw bones are symmetrically developed;
e) A patient is motivated to undergo treatment;
f) Regular oral hygiene and sanitation;
g) The dental alveolar pathology is the same in both jaws;
h) There is consent in the form of a written contract.

The following patients were not included in the study.

a) With tooth impaction;
b) With unformed roots of second M on both jaws;
c) With pathologies of the temporomandibular joint;
d) With refusal to remove third molars;
e) Who did not sign a consent to participate in the study.

The study was conducted in accordance with generally accepted norms of ethics and morality, and all patients were guaranteed anonymity and confidentiality of the information obtained. The research received approval from the Ethics Committee of Baoding Central Hospital.

### 2.3 Study protocol and design

The diagnosis was made based on the clinical examination, photometry of the face, teeth, anthropometry (digital and plaster models), X-ray and cephalometric findings. The patients were then divided into two equal groups of 20 based on the results. The first group consisted of patients treated with Experience braces and the second group with Invisalign aligners. Microimplants were also employed in both cases for all patients. Microimplants, also known as Temporary Anchorage Devices (TADs), were used as supplementary anchorage points to provide additional support and control over tooth and jaw movement. These microimplants were strategically placed in specific locations on the jaws or in the interdental region to achieve optimal control over tooth movement and create additional support. They were utilized to enhance fixation or support orthodontic appliances such as braces or aligners. In cases involving braces and aligners, microimplants (TADs) are typically positioned at various locations to ensure adequate support and control over tooth and jaw movement. However, the precise placement of microimplants may vary based on individual patient characteristics and treatment objectives.

Braces:

Microimplants can be installed in the alveolar process region (alveolar bone), providing support for tooth movement in a specific direction.

They can also be placed in interdental areas, offering an additional anchorage point to control jaw movement in a specific direction.

Microimplants can be placed in the palatal (palatine) bone or the buccal (cheek) bone, depending on the requirements for controlling tooth and jaw movement.

Aligners (e.g., Invisalign):

In the case of aligners, microimplants can be positioned in the same regions as for braces to provide additional support and control over tooth and jaw movement.

The precise placement of microimplants depends on the treatment plan and specific requirements of each case.

The placement of microimplants needs to be accurate and tailored to each patient to ensure effective treatment.

Example of Assessment and Treatment Protocol.

Step 1: Clinical Evaluation and Diagnosis

In this stage, the orthodontist (a dentist specializing in orthodontics) conducts a clinical evaluation of the patient, including an examination of the teeth, jaws, and facial structures. Additionally, photographs of the patient are taken, and radiographs are obtained to gather more detailed information about the tooth positions and skeletal structure.

Step 2: Model Fabrication and Impressions

The orthodontist creates plaster models of the patient’s teeth and jaws using impressions taken from the patient’s mouth. These models aid the orthodontist in gaining a better understanding of the current tooth positions and evaluating the required changes to achieve optimal alignment and occlusion.

Step 3: Analysis and Treatment Planning

The orthodontist analyzes the models, radiographs, and clinical data to determine the optimal treatment plan. Depending on the complexity of the case and desired outcomes, the orthodontist may consider two primary options: braces or aligners (e.g., Invisalign).

Step 4: Selection of Appropriate Appliances

Based on the analysis and treatment planning, the orthodontist selects the most suitable appliances for the patient. Braces are utilized for more intricate cases that require precise control over tooth movement. Aligners are preferred for less complex cases and patients who favour discreet treatment options.

Step 5: Development of an Individualized Treatment Plan

Based on the selected appliances and desired outcomes, the orthodontist formulates an individualized treatment plan for each patient. This plan encompasses the necessary steps to be undertaken to achieve the desired tooth alignment and occlusion.

Step 6: Treatment and Monitoring

Following the formulation of the treatment plan, the process of applying braces or utilizing aligners commences. Throughout the treatment, the orthodontist regularly monitors the progress and makes adjustments as needed to achieve optimal outcomes.

Step 7: Treatment Conclusion and Retention

Upon completion of the treatment with braces or aligners, the patient enters the retention phase. During this period, the patient wears retainers to preserve the achieved results and prevent the teeth from reverting to their original positions.

The clinical examination included the recording of complaints, the study of medical history (incl. dental), also the evaluation of the face, soft tissues of the mouth, and teeth. Based on the data obtained, a primary diagnosis was made. The Engel classification was used in this study. Dental photography was also used before, during, and after therapy. This was necessary to study the parameters of the face and the degree of teeth occlusion. Jaw impressions were taken before treatment, using Hydrogum alginate, after which plaster models were made. Group 2 patients were also asked to undergo a 3D dental scan.

Visualization of orthodontic treatment planning and the generation of 3D models of teeth and jaws through 3D scanning represent crucial tools in contemporary orthodontics.

Visualization of Planning.

a. Clinical Images: To begin with, the orthodontist conducts a clinical evaluation of the patient, including capturing facial and dental photographs. Clinical images assist the orthodontist in analyzing the facial features, occlusion, and tooth positions of the patient.
b. Radiographs: The orthodontist takes radiographs, such as panoramic radiographs or lateral cephalograms, to obtain more detailed information about the skeletal structure and tooth positions.
c. Fabrication of Plaster Models: Following the acquisition of impressions from the patient, the orthodontist fabricates plaster models of the teeth and jaws. These models aid the orthodontist in conducting a more precise analysis of the current tooth positions and planning their movement.
d. Digital Visualization: In modern orthodontic practice, specialized software can be utilized for digital treatment planning visualization. With the assistance of such software, the orthodontist can create virtual 3D models of the patient’s teeth and jaws.

Creation of 3D Models Using 3D Scanning:

a. Teeth and Jaw Scanning: To generate 3D models of the patient’s teeth and jaws, the orthodontist employs a 3D scanner. The 3D scanner is a device that traverses over the teeth and jaws, creating a precise 3D surface reconstruction.
b. Data Processing: After scanning, the acquired data undergoes processing through specialized software. The software analyzes the scanned data and generates an accurate 3D model of the patient’s teeth and jaws.
c. Virtual Planning: The obtained 3D models of the teeth and jaws can be employed for virtual orthodontic treatment planning. The orthodontist can manipulate the teeth on the 3D model and visualize the anticipated treatment outcomes.
d. Custom Appliance Fabrication: The obtained 3D models can be utilized for fabricating customized appliances, such as braces or aligners, which will be employed during treatment.

3D scanning and treatment planning visualization enable the orthodontist to conduct more precise and predictable treatment, thereby enhancing the quality of outcomes and patient convenience. These technologies are actively employed in modern orthodontic practice, facilitating a more efficient and personalized treatment approach for each patient.

### 2.4 Research methods

Measurement of Tooth Movement During Treatment:

Clinical Examination: Involves visual assessment of tooth positions and their relationships within the oral cavity. The orthodontist analyzes tooth alignment, displacements, tilting, rotations, and other changes.

Anthropometry: Measurement of various dental parameters on plaster models, such as distances, angles, dental arch width, etc., using methods such as Pont’s, Korkhaus, and Nance.

Each of these methods is designed to address specific objectives and provides information on different aspects of the dental and jaw system.

Pont’s Method:

Objective: Measurement of proportions of anterior teeth, including their height and width.

Application: Pont’s method is widely utilized for assessing the sizes of anterior teeth and their interrelationships. It can be valuable for diagnosis and treatment planning in the reconstruction of the anterior segment of the dental arch, including orthodontic treatment and dental restoration.

Korkhaus: Method

Objective: Measurement of angles between different teeth and dental arches.

Application: The Korkhaus method is commonly employed to assess angular characteristics of teeth, such as inclinations, overjets, protrusions, and retrusion. These measurements can be valuable for diagnosing malocclusions, orthodontic planning, and occlusal evaluation.

Nance Method.

Objective: Measurement of dental arch width and interarch distance

Application: The Nance method allows for the determination of dental arch width, which can be crucial in orthodontic treatment planning, particularly when the expansion of the arches is required to create space for proper tooth positioning.

A research study that utilizes these methods simultaneously possesses a comprehensive nature and aims to explore various aspects of the dental and jaw system. For instance, such a study may analyze not only the sizes of anterior teeth (using Pont’s method) but also their angular characteristics and dental arch width (using the Korkhaus and Nance methods, respectively). This approach allows for a more comprehensive understanding of the patient’s dental and jaw system and can be beneficial for diagnosis, treatment planning, and outcome evaluation.

Radiography: Utilizing various radiographic methods, such as computed tomography (CT), panoramic radiography, and lateral and frontal cephalometric radiography, to measure the angulation and displacement of teeth.

Assessment of treatment effectiveness

Comparison of Diagnostic Baseline Data: Comparing initial parameters and diagnosis with post-treatment results to determine the degree of changes and achievement of treatment goals.

Evaluation of Tooth Movement Range: Measuring the distance that teeth have been displaced during the treatment period.

Assessment of Occlusal Quality: Evaluating tooth contacts during occlusion to determine the level of alignment and dental arch closure.

Evaluation of Degree of Molar Distalization: Assessing the movement of molars towards the posterior part of the jaw (distalization) using orthopantomography and other radiographic methods.

Influence of Other Factors on Treatment Outcomes:

Control Groups: In our study, two groups of patients were included, one treated with braces and the other with aligners. Comparing treatment outcomes in different groups allows us to determine which treatment method was more effective for various objectives.

Consideration of External Factors: Age, gender, severity of dental and jaw anomalies, and other factors may influence treatment outcomes.

To make an accurate diagnosis and adequately proceed with therapy planning, the following X-ray methods were used: 1) computed tomography and the orthopaedic tomography method; 2) MRI (magnetic resonance imaging). The OHI-s index and Russell index were applied in the analysis of the oral cavity tissue state for the patients who participated in the study.

To ensure the reliability and accuracy of the results in our study, an assessment of measurement errors for the utilized methods was conducted. This aspect plays a crucial role in confirming the validity of the obtained data.

The assessment of measurement errors was conducted for each measured parameter on dental plaster models using the following approaches:

Standardization of Measurement Instruments: Before conducting measurements, standardized instruments were utilized and calibrated, which allowed for minimizing errors associated with the measurement tools.

Inter-observer Reliability Tests: To evaluate the interpretation of measurement results by different researchers, inter-observer reliability tests were performed. Several researchers independently measured the parameters on some samples, and then the correlation coefficient between their measurements was calculated.

Intra-observer Reliability Tests: The same researcher conducted repeated measurements of the same dental samples with a time interval between measurements. This allowed calculating the coefficient of correlation within one observer and determining the degree of measurement repeatability. In our study, the treatment procedures were carried out by licensed and experienced orthodontists with high qualifications in the field of orthodontics. In all cases, the treatment was performed by the same operators to minimize systematic errors and ensure consistency throughout the therapy.

Statistical Analysis: To calculate and analyze measurement errors, appropriate statistical methods such as the Intraclass Correlation Coefficient (ICC) and Bland-Altman plots were utilized. These methods were employed to assess the agreement between observers and to evaluate the measurement repeatability.

The obtained data were processed in the program Statistica (version 10). Student’s t-criterion for independent samples was used to reveal reliable differences. In case the sample was the same, for example, the group before and after therapy, paired Student’s t-criterion was used. The Student’s t-test for independent samples is used to compare the means of two different groups. In our study, there were two groups of patients: Group 1, which underwent treatment with braces, and Group 2, which was treated using aligners. These groups are independent samples, as each patient belongs to only one of them, and the results of one patient are not dependent on the results of others.

The application of the Student’s t-test for independent samples involved comparing the mean values of the measured parameters between the groups. For instance, it was possible to compare the mean value of the degree of molar distalization in the upper jaw between the group with braces and the group with aligners.

After conducting the statistical analysis using the Student’s t-test, the obtained *p*-value was obtained. The *p*-value represents the probability of observing the observed difference between the mean values of the two groups if, in reality, there is no such difference in the population.

If the *p*-value is less than or equal to the predetermined level of significance α (in our case, α = 0.05), then the difference between the mean values is considered statistically significant. Consequently, the null hypothesis of equal means is rejected, and we can infer the presence of statistically significant differences between the groups. Differences were significant at *p* ≤ 0.05.

### 2.5 Ethical issues

During the research, informed consent was obtained from each participant. Participants were provided with comprehensive information about the objectives and procedures of the study, potential risks and benefits, as well as the option to withdraw from participation at any time without any negative consequences.

All data collected from the participants were processed with strict adherence to confidentiality. The personal information of the participants was anonymized, and access to the data was restricted to members of the research team only.

The study was conducted by the principles and standards of ethics in scientific research. This included respecting the rights and dignity of the participants, preventing potential harm, and safeguarding the interests of the participants.

The research was approved by the Research Committee (Ethics Committee of Peking University Medical University, protocol number 440), adhering to the principles of scientific ethics and protecting the rights and safety of the participants.

All research findings were presented with honesty and objectivity. All methods used in the study were described in detail to allow replication by other researchers.

The researchers took full responsibility for conducting the study by ethical requirements and ensuring the safety and wellbeing of the participants.

Researchers avoided any conflicts of interest that could have influenced the objectivity and credibility of the research results.

## 3 Results

For the patients who participated in the study, the state of their teeth and jaws was examined, using methods of clinical diagnosis, as well as the calculation of jaw structure models, methods of radiography and functional studies. During the study, patients with diagnoses meeting the inclusion criteria were established.

Anthropometric methods yielded results on the dynamics of width parameters for the lower and upper dentitions. These parameters were calculated for first premolars and first permanent molars (Table 1). It was found that there was an insignificant degree of narrowing of the upper row (0.7%–2.3%) in relation to the norm in the premolars zone and 2.2%–3.9% in the zone of first molars.

**TABLE 1:** Parameters of anthropometry of the upper row (zone of first premolars and first molars).

Indicators		Values of the norm	Parameters before therapy
Maxillary anterior teeth width (according to the Pont’s method)	zone of 1st premolars	35.4 ± 2.3	34.6 ± 2.6
	zone of 1st molars	46.2 ± 2.8	45.8 ± 2.6
Maxillary anterior teeth length (according to Korkhaus methodology)		17.1 ± 1.2	16.6 ± 1.2
Indicators of mesialization pathology of 1st molars (upper jaw)		-	4.7 ± 0.2

The parameters of the anterior maxillary length decreased by 3.2% in relation to the norm. The pathology parameters of the lateral group were within the range of 4.5–4.9 mm. The angulation parameters of the lateral group about the base of the maxilla were close to normal (Table 2).

**TABLE 2:** Indicators of mesial inclination in the lateral group (upper jaw) in relation to the base of the upper jaw.

Angulation indices in relation to the plane (upper jaw)	Norm indicators (according to Weber), degrees	Patient readings, degrees
1st premolars	92.3 ± 2.5	94.2 ± 1.3
1st molars	67.6 ± 2.1	79.0 ± 0.8

**FIGURE 1:**
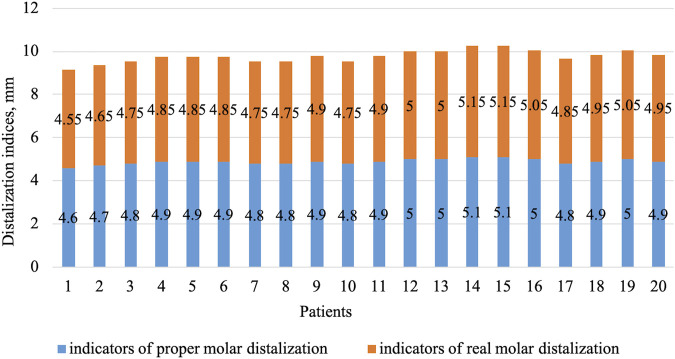
Photographs after completion of braces treatment: **(A)** right side view; **(B)** left side; **(C)** maxilla from above; **(D)** mandible from above; **(E)** front view.

The mesial inclination values that did not correspond to the norm according to Weber were in all patients who participated in the study. Angulation degrees deviated by 16% from the normal values. During the analysis of patients’ radiographs, the level of inclination was assessed for the central incisors in the upper and lower rows in relation to the planes of the dorsal and mandibular axes. Indicators ∠Ui/Spp in all patients varied and ranged from 133 to 124°. The linear parameters of the examined jaws corresponded to the norm. Based on the findings, the patients were diagnosed with a distal type of occlusion of the first subclass. Derived from this, a treatment plan was devised, encompassing tasks like molar distalization and creating free space behind the canines. This allowed for potential adjustments to the maxillary angle using aligners or braces. When evaluating the width of the upper dentition after distalization, braces showed a 3.6% expansion in first molars in relation to the norm and 5.4% in relation to the pre-treatment values. For first premolars, the same figures were 4.5% and 3.2% (Table 3; Figure 1).

**TABLE 3:** Pre- and post-therapy dentition width values in group 1 patients (braces), in millimetres.

Parameter name		Indicators of the norm	Before therapy	After therapy	The magnitude of the difference between the values before and after therapy
The values of the width of dentition in the upper jaw (according to the Pont method)	1st premolars	35.5 ± 2.2	34.8 ± 2.6	37.2 ± 1.7	2.4 ± 0.6
	1st molars	46.2 ± 2.8	45.8 ± 2.6	48.2 ± 2.7	2.4 ± 0.1

**FIGURE 2:**
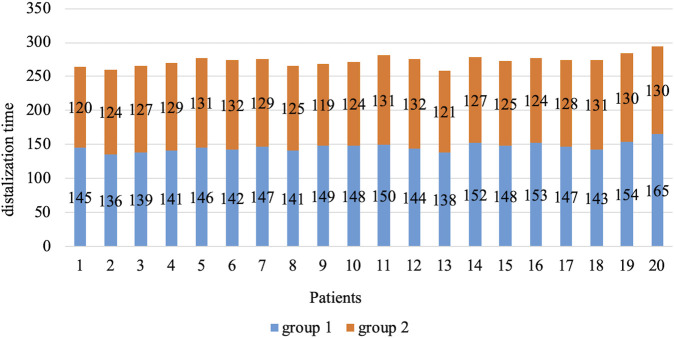
Results of using Invisalin aligners. **(A)**. Movement of 2 M. **(B)**. Movement of 1 M.

For patients in group 2 (Invisalign aligners), no change was found in the overexpansion of the upper dentition for first molars and first premolars (Table 4; Figure 2). Relative to the start of therapy, there were 4% and 5% changes in overexpansion rates for first premolars and first molars, respectively.

**TABLE 4:** Pre- and post-treatment values for patients in group 2 (Invisalign aligners), in millimetres.

Parameter name		Indicators of the norm	Before therapy	After therapy	The magnitude of the difference between the values before and after therapy
The values of the width of dentition in the upper jaw (according to the Pont method)	1st premolars	36.0 ± 1.5	34.6 ± 2.3	36.2 ± 1.7	1.6 ± 0.6
	1st molars	47.6 ± 1.6	45.6 ± 2.2	47.7 ± 1.9	2.1 ± 0.3

**FIGURE 3:**
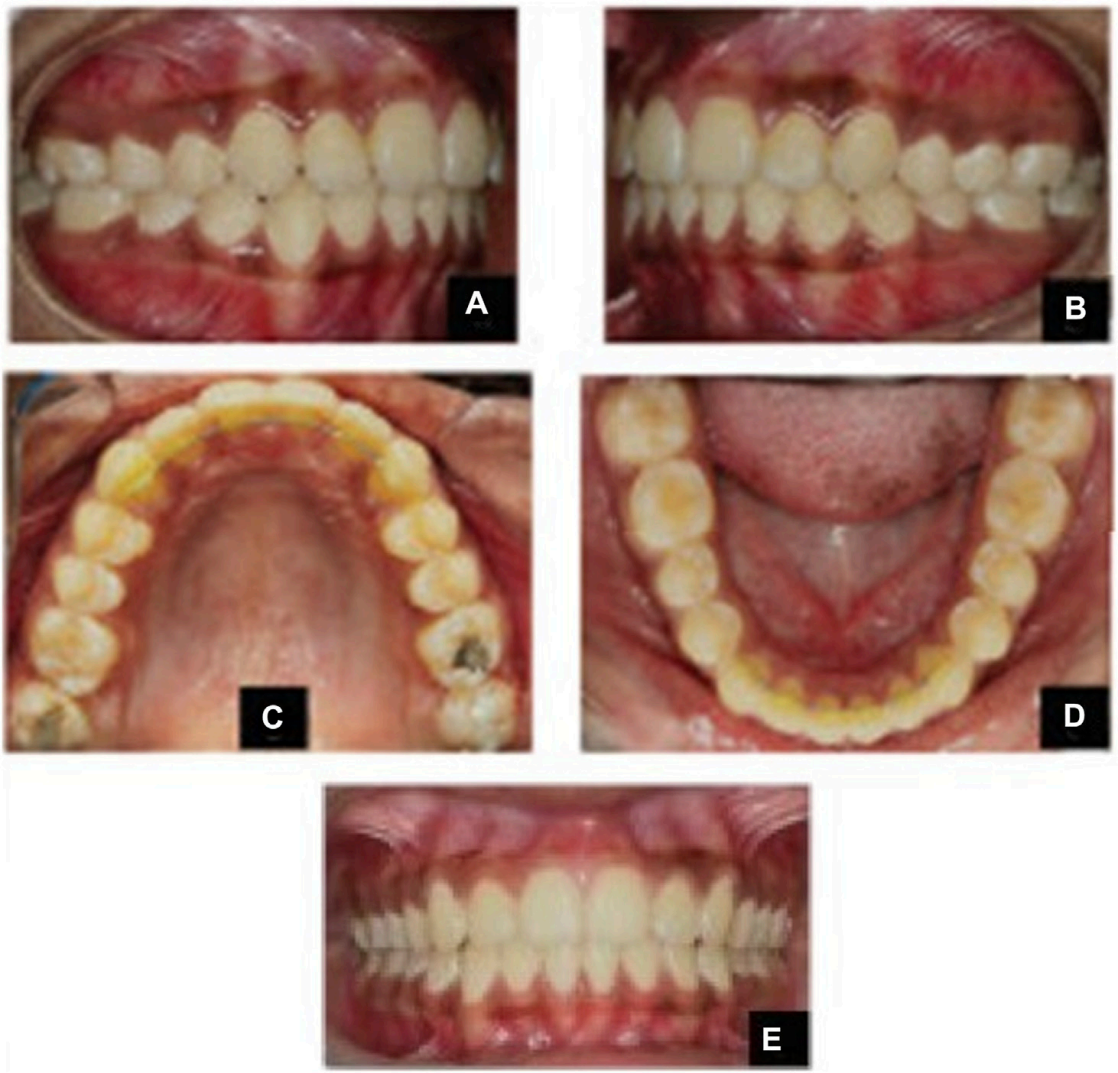
Indices of real and desired degree of distalization for patients in group 1 (first molars).

**FIGURE 4:**
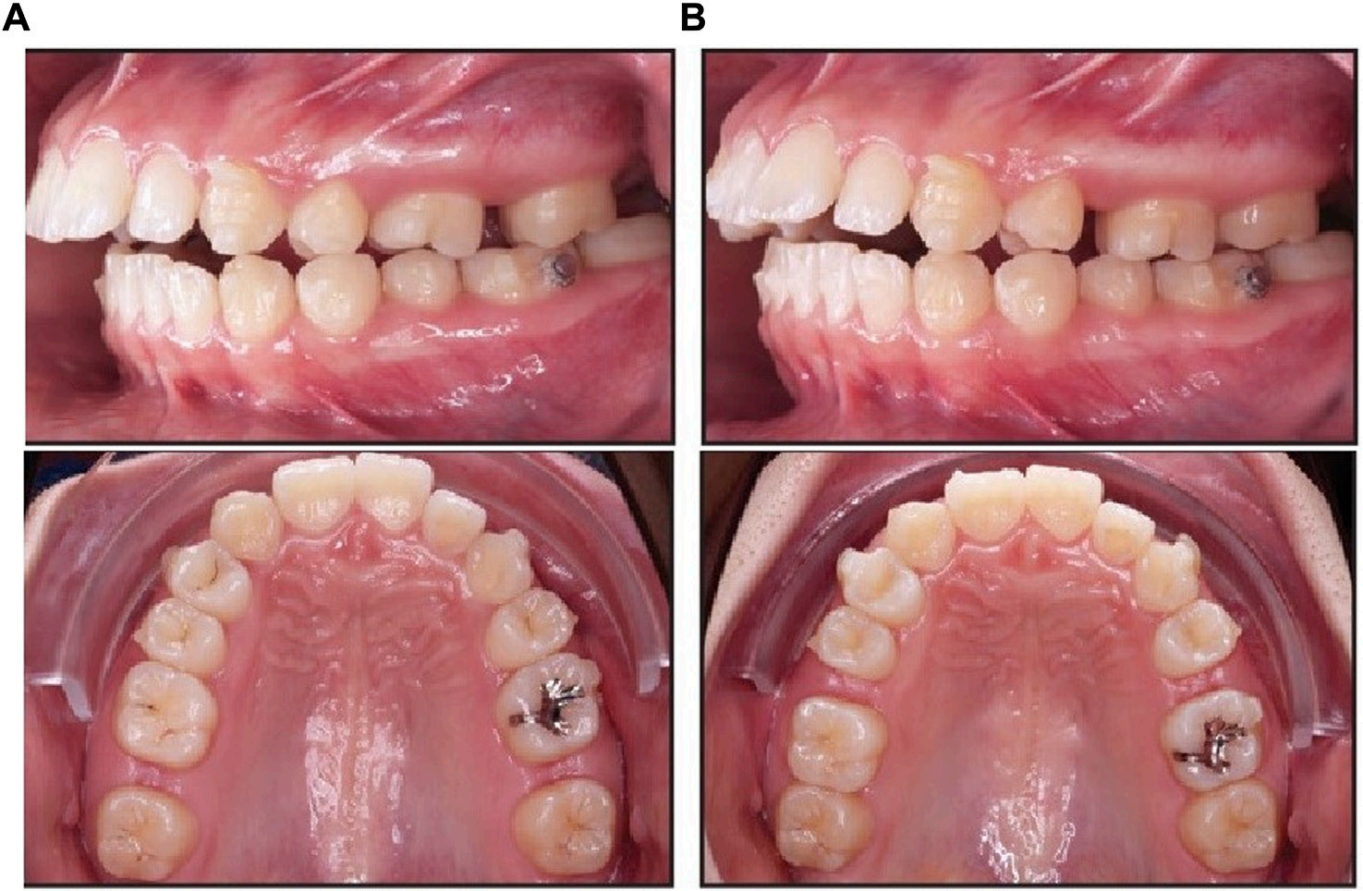
Indices of real and desired degree of distalization for group 2 patients (first molars).

Based on the data obtained on the clinical parameters, it can be argued that during therapy in patients from group 2, there was an approximation of dentition width parameters to the norm in the lateral teeth group. This is because the program included control of the degree of expansion in first premolars and first molars. Braces do not offer the same level of control, resulting in overextension occurring in the lateral group. No statistically reliable differences were established. At the same time, differences in the indices of distalization of first molars in the upper jaw between the two groups of patients were established (Table 5; Figure 3; Figure 4). Before the start of therapy, the mesial displacement index was 4.8 mm. After therapy, it was found that the use of aligners and microimplants removed the shift. For all patients with aligners, the shift reduction was 100% and the error was less than 0.5% due to the inherent features of 3D scanning. For patients in group 1, there was a 92% decrease in shear relative to the required one, and the remaining patients had a 5% greater distalization of the premolars than normal.

**TABLE 5:** Changes in the distal displacement of first molars in patients from groups 1 and 2 (in millimeters).

Group	Before therapy	After therapy	Average value of distalization parameter
1	4.7 ± 0.4	0 ± 0.4	4.8 ± 0.6
2	4.8 ± 0.3	0 ± 0.05	4.8 ± 0.3

**FIGURE 5:**
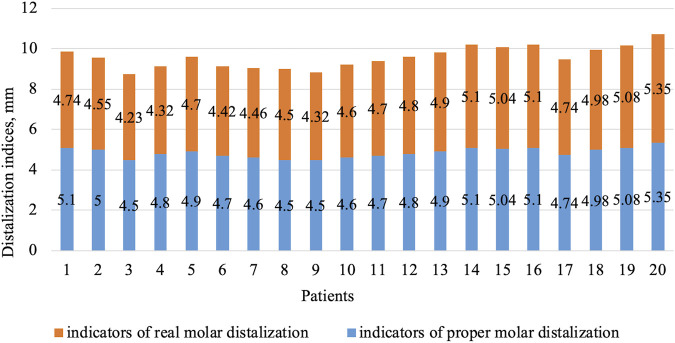
Time taken for distalization of first molars in groups 1 and 2.

It follows that prediction using a computer program is more effective because it is possible to obtain the desired and real volume of distalization, which was not observed in patients from group 1. No statistical differences were found. Patients in groups 1 and 2 visited an orthodontic specialist once every 14 days. This was necessary to change the tractions between the implants on one side and the braces or aligners on the other. All 40 patients had an identical traction step with the same tension force. Next, the number of days required for a complete degree of distalization was calculated, as well as the rate of the same parameter for first molars (Table 6; Figure 5).

**TABLE 6:** Parameters of volume (I, mm), time (II, days) and rate (III, mm per day) of distalization in groups 1 and 2.

Group	I	II	III
1	4.78 ± 0.57	151 ± 12	0.0327 ± 0.0025*
2	4.86 ± 0.27	125 ± 6	0.040 ± 0.001*

**Note:** *- reliable at р≤0.01.

From the data obtained, it follows that with the same parameters of force, volume, and conditions, it took 19 days less to perform distalization of first molars in group 2 than in group 1, which is 17%. For group 2, the movement of first molars was 0.0065 mm on each day, which was 17% higher than in group 1. The patient’s oral hygiene was examined using two indices shown in Table 7.

**TABLE 7:** Index values before and after therapy.

Group	OHI-S index		Russell index	
	Before therapy	After therapy	Before therapy	After therapy
Group 1	0.5 ± 0.2*	1.2 ± 0.2*	0.5 ± 0.2	0.9 ± 0.3
Group 2	0.5 ± 0.3	0.6 ± 0.2*	0.4 ± 0.2	0.8 ± 0.3

**Note:** *- reliable at р≤0.05.

In group 2, the values of both indices were the same as in the normal range (Table 7). Patients in group 1 had a diagnosis of K05.1 which corresponds to a satisfactory level of hygiene. This suggests that Invisalign aligners may lead to improved oral hygiene results for patients as they have the advantage of being removable, enabling easier monitoring of their oral hygiene practices. At the same time, the periodontal status worsened in both groups due to the application of bound force. This is also because aligners exert pressure on the gums. Thus, therapy with Invisalign aligners showed better and more comfortable results for patients compared to braces.

### 3.1 Summary of findings

In this study, the parameters and treatment outcomes of patients participating in the research were analyzed. The patients underwent clinical examination, and anthropometric, radiographic, and functional methods were applied to study the condition of teeth and jaws. Diagnoses were established based on inclusive criteria.

The results of anthropometric measurements revealed the dynamics of width parameters in the upper and lower dental arches. Values were determined for first premolars and first permanent molars. It was observed that the level of narrowing in the upper arch was insignificant (0.7%–2.3%) in the premolar region and amounted to 2.2%–3.9% in the first molar region.

The length parameters of the anterior segment of the upper jaw decreased by 3.2% relative to the norm. Pathology indicators of the lateral group were within the range of 4.5–4.9 mm. Angular parameters of the lateral group about the base of the upper jaw were close to the norm.

Additionally, it was found that the angular values of mesial inclination did not conform to Weber’s norm in all the patients participating in the study. The degree of angular deviation amounted to 16% of the normal values. After analyzing the radiographs of the patients, the degree of inclination of the upper and lower front incisors relative to the planes of the posterior and lower axes was assessed. For all patients, the values of ∠Ui/Spp ranged from 133 to 124°. Linear parameters of the investigated jaws corresponded to the norm.

Based on the identified results, patients were diagnosed with a distal type of Class I malocclusion. This served as the basis for developing a therapeutic plan, including tasks for molar distalization and space regaining behind the canines to alter the angle in the upper jaw using aligners or braces.

Subsequently, the results of upper dental arch expansion after distalization with braces and aligners were analyzed. For patients with braces, an expansion of 3.6% for first molars and 4.5% for first premolars compared to the norm, and 5.4% and 3.2% respectively compared to the initial values, was observed. In contrast, no changes in the upper dental arch transverse dimension were detected for patients in group 2 (aligners).

The data obtained from the study suggests that treatment utilizing computer programs is more effective in achieving the desired and actual extent of molar distalization. Additionally, it is noted that patients treated with aligners experienced more comfortable outcomes and exhibited a better level of oral hygiene compared to patients wearing braces. This observation can be attributed to the aligners’ removable nature, which facilitates easier hygiene maintenance.

### 3.2 Study limitations

The size of the study groups may constitute a significant factor in determining the statistical power and level of significance of our research.

The planning of the group sizes was conducted considering the available resources and constraints, such as patient availability and the duration of the study. Nevertheless, our utmost efforts were directed towards ensuring the reliability and validity of the results to make our investigation as informative and valuable as possible.

Despite the relatively small size of the study groups, our research was able to detect statistically significant differences and achieve a level of significance set at *p* ≤ 0.05. We also conducted a power analysis, confirming that the power level reached approximately 85%, indicating our ability to detect real differences if they exist in the population.

Nonetheless, we recognize the importance of increasing the sample size and enhancing the study’s power to more accurately detect differences between the groups and validate our findings. Future research endeavours may consider enrolling a larger number of participants to provide additional support for our conclusions.

## 4 Discussion

The search for the optimal solution to the problem of dental anomalies is an ongoing task of research teams. Progress is being made in refining techniques, instruments, and apparatus, yet despite these advancements, a definitive solution to the problem remains elusive (Laishram and Thongam, 2022). Previously, in the presence of anterior crowding, in the presence of dental-alveolar abnormalities, the most common solution was to remove premolars (Bazarova et al., 2021; Yeslyamgaliyeva et al., 2021). Removal was performed by extraction, one per segment (Genest-Beucher et al., 2018). At the same time, another problem subsequently arose: how to close the gaps after extraction. Bodily movement type occurred among the masticatory teeth (Soni, 2017). A consequence of this was the replacement and creation of pockets according to bodily and lingual root/crown tipping movement types. Food inevitably fell into such pockets (Smirnova et al., 2012; Karkhanechi et al., 2013; Gildeeva et al., 2019). After the development and active use of aligners, it has been possible to increase the rate of treatment, as demonstrated in the present work. In addition, as shown in the present work and other works, it is possible to perform directed and precise distalization without the use of additional means other than attachments (Simon et al., 2014). Aligners do not traumatize the oral cavity, which is one of their key differences from braces. This is because the surface of the aligner is smooth and cannot harm the oral mucosa (Rajput et al., 2023). Another difference between aligners and braces is that their locks are fixed at control points, not on all teeth (Megat Abdul Wahab et al., 2012). In addition to the advantages of aligners shown in the present work, they also have their disadvantages. The present study included only patients without cavities and with a sanitized mouth, which is a limitation not only of the study but also of the method in general (Moshiri et al., 2013). Filled teeth are also desirable to be filed and removed from contact with the aligner, or to order new aligners, which will affect the cost and timing of therapy (Buschang et al., 2014). In the case of some patients, the development of disocclusion is allowed, with a total aligner thickness of 1.2 mm on both jaws. In this case, the contact occurs in 2 places (on the canines) (Huang and Huang, 2022). Finally, the formation of the gaps may result in pieces of food getting stuck, and bleeding of the gums may be detected. As a result, pockets of pathological origin may arise (Partouche et al., 2022). These problems with the use of aligners are a consequence of inaccuracy in their production technology. To eliminate the problem, two ways can be followed (Huang and Huang, 2022). The first is the separation (1 time in relation to 8–12 steps). Due to the fact that there is a thinning of the enamel layer and the development of high levels of sensitivity, and the risk of caries development, it can lead to a deterioration of a patient’s dental health. The second way is to take impressions at every stage of the therapy when it is not possible to move the teeth without doing so. This can affect the price and the timing of the therapy. The use of Dynamic Physical Model technology involves the analysis of radiographs, facial and oral images. After obtaining the final impression, a plaster cast is created, and a dynamic pin is utilized to delineate the contours of each tooth. The model is divided into individual teeth. After activating the pins, they are moved in increments of 0.25–0.5 mm. The aligner is then pressed to ensure its high level of accuracy (Pettersson, 2011). As shown above, aligners have advantages and disadvantages. At the same time, due to their safety and practicality, they have found widespread use in dental practice. One positive aspect for patients is that aligners offer invisibility, accuracy, and a customized fit. Reports indicate that aligners enable the correction of teeth position parameters and the resolution of occlusion anomalies (sagittal plane) irrespective of their complexity. Moreover, some authors highlight the potential use of aligners not only in adults but also in young adolescents (Sauer et al., 2023).

As a practical recommendation stemming from the study’s findings, the authors propose the following approach: when deciding on the appropriate treatment for patients diagnosed with mesial lateral group displacement, both the underlying causes of the condition and potential consequences, such as periodontitis, should be carefully considered. In addition, the presence of third molars as rudiments, as well as the size of the retromolar space, must be taken into consideration. If molar distalization with aligners is carried out with microimplants, it is of the bodily type if horizontal and vertical attachments are placed on the teeth to be moved. The size of the attachments should be 3 mm long, 1 mm wide, and 0.9 mm thick. The step size per aligner is up to 0.47 mm, and the duration of wearing aligners is 14 days.

## 5 Conclusion

There were no significant differences in molar distalization between braces (group 1) and aligners (group 2). It took 17% less time for aligners to distalize first molars. Significant differences were obtained in the rate of distalization: in group 2 it was 6.5 µm greater at 1 day, 0.195 at 30 days. This was 17% higher than in group 1 patients. Aligner therapy made it possible to completely reduce the effect of the mesial shift. The process of molar distalization in patients in group 2 was of the bodily type but without distal angulation. For patients from group 1, there was a 10.6% increase in the distal angulation of first molars after therapy. The distalization of first molars in group 1 was 93%; another portion of patients in this group had distalization greater than necessary by 5%. Group 1 patients showed a 3.5% increase in the width of first molars above the normal parameters. There was an increase in the Russell index by half in both groups of patients compared to the indicators before the therapy. The method of distalization did not affect the decrease of the paradontal index values. The OHI-S index was found to increase by 58.5% compared to the pre-treatment values in group 1, whereas in group 2 the index increased by 15%.

For molar distalization using aligners and microimplants, a horizontal rectangular attachment should be placed on the distal tooth 1 mm higher on the cheek side in relation to the equator. For one aligner the movement should not be more than 0.47 mm (once every 14 days per 1 aligner). Microimplants are placed in the retromolar area to reinforce bodily movement. The traction force parameters should be between 1.5 and 3.0 N.

Prospects for further research:

Future investigations could increase the number of participants and expand the sample size, thereby enhancing the statistical power and generalizability of the findings.

Conducting comparative studies with other orthodontic anomaly treatment methods is crucial to determine which approaches may yield the best outcomes for specific patient types.

Long-term follow-up studies on patients would provide valuable insights into the stability of results and the long-term consequences of using braces and aligners.

The practical significance of this research lies in its potential to optimize treatment approaches. The study’s outcomes may assist orthodontists in selecting the most effective and suitable treatment methods for each patient, considering their characteristics and needs.

## Data Availability

The original contributions presented in the study are included in the article/supplementary material, further inquiries can be directed to the corresponding author.
